# Systems approaches for localising the SDGs: co-production of place-based case studies

**DOI:** 10.1186/s12992-019-0527-1

**Published:** 2019-12-18

**Authors:** David T. Tan, José Gabriel Siri, Yi Gong, Benjamin Ong, Shiang Cheng Lim, Brian H. MacGillivray, Terry Marsden

**Affiliations:** 1 International Institute for Global Health, United Nations University, Kuala Lumpur, Malaysia; 2 Sustainable Places Research Institute, Cardiff University, Cardiff, Wales, UK; 3 Urban Biodiversity Initiative, Kuala Lumpur, Malaysia

**Keywords:** Systems approaches, Systems thinking, Place-based approaches, Placially explicit, Localisation, Co-production, SDGs

## Abstract

**Background:**

Localisation is a pervasive challenge in achieving sustainable development. Contextual particularities may render generalized strategies to achieve the Sustainable Development Goals (SDGs) unfeasible, impractical, or ineffective. Furthermore, many localities are resource- and data-poor, limiting applicability of the global SDG indicator framework. Tools to enable local actors to make sense of complex problems, communicate this understanding, and act accordingly hold promise in their ability to improve results.

**Aim:**

Systems approaches can help characterise local causal systems, identify useful leverage points, and foster participation needed to localise and catalyse development action. Critically, such efforts must be deeply rooted in place, involving local actors in mapping decision-processes and causation within local physical, social and policy environments. Given that each place has a unique geographical or spatial extent and therein lies its unique characters and problems, we term these activities “placially explicit.” We describe and reflect on a process used to develop placially explicit, systems-based (PESB) case studies on issues that intersect with and impact urban health and wellbeing, addressing the perspectives of various actors to produce place-based models and insights that are useful for SDG localisation.

**Methods:**

Seven case studies were co-produced by one or more Partners with place-based knowledge of the case study issue and a Systems Thinker. In each case, joint delineation of an appropriate framing was followed by iterative dialogue cycles to uncover key contextual factors, with attention to institutional and societal structures and paradigms and the motivations and constraints of other actors. Casual loop diagrams (CLDs) were iteratively developed to capture complex narratives in a simple visual way.

**Results:**

Case study development facilitated transfer of local knowledge and development of systems thinking capacity. Partners reported new insights, including a shifting of problem frames and corresponding solution spaces to higher systems levels. Such changes led partners to re-evaluate their roles and goals, and thence to new actions and strategies. CLD-based narratives also proved useful in ongoing communications.

**Conclusion:**

Co-production of PESB case studies are a useful component of transdisciplinary toolsets for local SDG implementation, building the capacity of local actors to explore complex problems, identify new solutions and indicators, and understand the systemic linkages inherent in SDG actions across sectors and scales.

## Background

### Translating SDGs into local contexts

The United Nations Sustainable Development Goals (SDGs) were adopted in 2015 as a globally agreed vision for advancing the conditions of people and planet while ensuring that no one is left behind. Because health is central to and deeply interlinked with sustainable development [1], progress toward these goals is essential for creating physical, social and policy environments that will sustain and enhance health and wellbeing. Realising such progress will require action at local scales; yet localisation involves more than just local application of high-level agendas. Rather, SDG localisation encompasses local agenda setting, decision-making, and process monitoring with locally adapted indicators, which together generate the ownership necessary for successful SDG implementation at local scale. It is thus critical that the apparatus of sustainable development focus on being more responsive and relevant to local needs and aspirations.

Substantial difficulties exist in translating high-level SDG goals for local contexts, as evidenced by the literature on gaps between global indicators and local needs and understandings. Global indicators enable comparison between contexts but may sacrifice local validity [2, 3] and the ability to motivate action by reflecting local values [4, 5]. Furthermore, global indicators may be unusable at local scales where data, or the resources and capacity to obtain such data, are unavailable [2, 3]. The necessary development of SDG indicators that match locally available needs, values, and capacity is hampered by the same resource and capacity deficits that limit local application of global indicators. Furthermore, responsibilities and expertise are vertically and horizontally fragmented. This fragmentation frequently isolates local actors, depriving them of support and empowerment and thereby limiting meaningful participation and ownership. This is problematic given that participation, rooted in place as an organising principle, is critical for connecting domains such as urban planning and health [6–8] that are critical for SDG action.

### Systems approaches for localisation

Despite shared emphases on contextual understanding and holistic approaches, systems thinking and place-based approaches have rarely, and only recently, been mentioned together in scientific literature [9–11]. They intersect in systems approaches [12–15], which are strategies for problem exploration, framing and solving that make use of systems thinking tools and methodologies in tandem with participatory engagement beyond the academy.

Systems thinking has been defined in various ways, with key elements including a consideration of interconnections, holistic rather than reductionist approaches, and exploration of dynamic and emergent behaviour arising from the action of feedback relationships [16]. Systems thinking provides tools for managing complexity by shifting problem frameworks from linear cause-effect interpretations toward an understanding of the larger context in which interventions might occur, how other actors might respond, and unintended consequences that might affect not only outcomes but also interventions themselves [17].

Place-based approaches are motivated by the idea that sustainability problems are often best understood by analysing human–environment interactions in specific locations and at relatively small scales. This is generally justified in terms of analytical tractability, or on the grounds that macro-scale approaches involve the sacrifice of process detail, or in the belief that human–environment interactions are strongly context-sensitive [18, 19]. An important critique of the local approach to sustainability issues is that action which is locally optimal may shift externalities to other scales, sectors, or locations. Systems thinking, with its emphasis on interconnectedness, can be a useful corrective to this.

Systems approaches make use of Causal Loop Diagrams (CLDs) and other systems thinking tools to enable participatory exploration of problems. In addressing local problems, a place-specific approach is critical, because problems manifest in unique ways in particular contexts. Although common contextual features are often shared across different settings, the interconnections and interdependencies between parts of systems (especially between people and environments) are often diverse, dynamic, and, most importantly, place-specific. Pre-existing social, cultural, economic, and environmental conditions in different places also play a significant role in determining the structure of causal chains [20]. Thus, lessons are not easily translated across contexts. Rather, efforts to address local problems—such as in SDG localisation—require a placially explicit understanding of the relationships and interconnections in that place.

In systems approaches, placially explicit understanding is achieved by engaging actors and stakeholders with systems tools for model- and narrative-building. These methodologies provide a common language that is a necessary part of any solution for overcoming disciplinary and organisational fragmentation and enabling diverse stakeholders to create shared narratives about important development issues [21]. A common language is critical to transdisciplinary work that integrates both academic researchers from different unrelated disciplines and non-academic participants to examine a common goal and create new knowledge and theory [22]. Narratives developed in transdisciplinary projects can advance localisation of the SDGs in various ways:

- By facilitating the creation and communication of holistic understandings of complex socio-ecological issues [23].
- By creating systems frameworks that are useful for evaluating likely leverage points and consequences of actions [24, 25], thus suggesting local SDG solutions and ways to parlay the SDGs into broader local development.
- By fostering the development of relationships among actors, thus providing a pathway for developing the local, intersubjective, value-based indicators advocated by Burford et al. [4, 5] and enabling the integrated multi-level partnerships that have been identified as one of the key drivers for SDGs localisation [26, 27].

Transdisciplinary systems thinking workshops have been a typical vehicle for building systems thinking capacity and applying systems methodologies to local problem exploration [28, 29]. Such workshops facilitate transdisciplinary action, often by training participants to overcome disciplinary barriers, and can thereby serve as a vehicle for systems approaches. However, while valuable, such workshops are often resource-intensive, and may require the convening of large groups of stakeholders. Here, we describe and reflect on a complementary capacity-building process: co-development of placially explicit, systems-based (PESB) case studies. Such studies are well-suited to meet intersecting challenges of SDG localisation in ways that improve health and wellbeing, especially in low-resource and low-capacity settings.

## Methods

While the urban environment, including land use and the built environment, is an important influence on health [30–34], the fields of urban planning and public health are limited in their interactions, the result of a long-standing divergence [6, 35]. Under the Systems Thinking and Place Based Methods for Healthier Malaysian Cities (SCHEMA) project, an effort to improve decision-making for urban health, PESB case studies were developed to demonstrate the value of systems approaches for improving understanding and developing narratives to address this and other such gaps, with the end goal of improved decision making. Simple CLDs were used to visually communicate the complex relationships among urban planning, public health, and other fields [21, 24]. These were combined with other written and visual elements to produce seven case studies (Table 1) aimed at policy-makers, which were launched at the 9th World Urban Forum (WUF9) in Kuala Lumpur, Malaysia.

**Table 1:** List of Case Studies

Case Study Description	Partners	Key Insights
1. Analysis of how policies and practices around school canteens interact based on observations of schools in a small township.	Anthropology researcher	Policies and practices surrounding school canteens with different goals (i.e., student nutrition, promotion of small businesses, and school finances) have been set independently of each other. While they make sense independently, they are incoherent together, undermining nutritional value of school canteen food.
2. Challenges in changing diets in Malaysia to combat rise in diabetes.	Health policy researchers	Health promotion through informational campaigns needs to be accompanied by strategies that address societal and environmental drivers of food consumption and physical activity.
3. Sustaining urban rejuvenation efforts in a financially limited locality.	Officers from an organisation funding and facilitating urban rejuvenation efforts	When urban rejuvenation efforts are coupled with a locally appropriate strategy for engaging communities and developing cross-sector partnerships, resources can be unlocked for maintaining improvements and initiating new efforts.
4. A university botanic garden’s challenge in maintaining conservation and education missions as university institutional priorities and funding shift.	University researcher and living laboratory programme officer	To maintain its mission, the botanic garden needs to re-evaluate who it considers as its key stakeholders and reorient its activities and focus to cultivate those relationships.
5. Competing paradigms within a university of the value of its undeveloped land, and the challenge of maintaining green spaces in urban centres.	University administration leader and living laboratory programme officer	To secure university green spaces, institutional paradigms and sustainable land use must be strengthened. To achieve this, linkages must be made between conservation and other core values and priorities the university holds.
6. Technological and community approaches to river clean-up and maintenance.	Civil society advocates and university researcher	Technology appears to offer predictable and easily implementable solutions to state and local authorities dealing with pollution issues. However, when this is the sole solution, communities are disempowered and become disengaged, strengthening paradigms that lead to increased pollution.
7. Bike-sharing as part of an integrated public-transit solution.	Private sector bike-sharing company	Barriers to cycling are lowered when there is a critical mass of cyclists such that driver-awareness and road infrastructure change to accommodate cycling. Bike-sharing companies can play a role in overcoming initial barriers such that this critical mass can be reached.

Case studies were co-produced by one or more partners with place-specific knowledge of the case study issue (“Partners”) and an expert in systems thinking (“Systems Thinker”). Table 1 briefly summarizes case studies and key insights. Partners were recruited via an open call and through professional networks. Partners included representatives from civil society organisations, policy researchers, academics, and the private sector. With one exception, Partners had no or minimal prior exposure to systems thinking.

Co-production of PESB case studies was designed to fully engage Partners in holistic problem definition and representation so that Partners retained control over the transfer of knowledge, often implicit or tacit, into the case study format [36]. The process began with selection of an appropriate framework for understanding the issue in question. Partners were provided with a short primer on CLDs, a sample case study, and a short set of guidelines. They then developed a 200-word abstract describing the problem and highlighting attempted or proposed solutions. The Systems Thinker followed up with Partners individually, via e-mail or a face-to-face meeting, with an iterative series of questions, based on principles in systems approaches, to map out the larger context in which the case study was embedded. Attention was given to institutional and societal structures and paradigms, as well as the motivations and constraints of other actors involved in the problem and/or solution.

Based on responses to initial questions, the Systems Thinker developed three to four candidate CLDs that attempted to create a conceptual model of causal linkages surrounding the problem and solution in a manner consistent with the Partner’s narrative. Partners were asked to identify what was correctly captured and what was left out, concluding with the selection of a preliminary CLD and corresponding problem frame that accurately represented the Partner’s understandings. The selected CLD went through several further iterations, informed by ongoing engagement. The CLDs were broken down into 3–4 stages of complexity. The simplest stage involved one or two key feedback loops, with further contextual detail added in subsequent stages. Potential systems-based interventions were usually added in the last stage. When the CLDs were finalised, Partners wrote the case study text, using the CLD stages as an outline. The Systems Thinker played an editorial role to ensure the text was consistent with and adequately explained the narrative portrayed in the CLDs. Contact time between the Systems Thinker and the Partners varied widely, averaging ten hours per case study on face-to-face time and written correspondence. The Systems Thinker spent around ten additional hours in developing CLDs and in the editorial role. Partners also spent between five to twenty hours in research and writing; the Partners who spent more time intended to use the content and analysis of the case studies in other aspects of their work.

## Results

Development of the PESB case studies facilitated a transfer of local knowledge from Partners to the Systems Thinker, and development of systems thinking capacity in the former. In five of the seven case studies, Partners engaged deeply with CLD development, giving substantive comments about CLD structure and variable naming. These partners reported new insights that changed the way they understood the highlighted issue. One factor that encouraged engagement was relevance to future work undertaken by the Partners, such as in Case Study #2, which the Partners undertook as background analysis for a study on relationships between fast food outlets and neighbourhood obesity. In the remaining two case studies Partners were more invested with developing a case study product than with the reflective process and were largely uncritical about the CLD representation. The sole case study developed with a for-profit private-sector partner, Case Study #7, was viewed by the Partner primarily as a communication tool for promoting bike-sharing and not as a learning activity.

The PESB case study methodology adopted here provided Partners with tools for describing a place in terms of feedback relationships and for understanding the origins of various consequences—desirable and undesirable. Initial case study abstracts by Partners were usually framed narrowly, with solutions presented as direct, linear responses to the problem. Through the process here described, Partners reframed their conceptualization of local challenges away from immediate problems, goals, and roles, instead mapping out the incentives, constraints, and goals of other actors within the system. In each case, the final problem frame was at a higher systems level—engaging with broader institutional and societal rules, values, and paradigms—than the original problem described in the abstract. For example, Case Study #3 on rejuvenating urban spaces began with sole focus on two rejuvenating efforts; the final writeup placed these efforts within the larger challenge of maintaining and expanding urban green spaces through partnerships between local authorities and private actors. The CLDs developed in the case studies provided conceptual models useful for hypothesizing about leverage points, causal pathways and theories of change, and for prioritising among evidence to be collected or generated. This resulted in proposed solutions at higher problem levels, understood in connection with larger causal pathways for change.

Improved understanding among Partners of the complex nature of their case studies has had real-world relevance as they continue to work and advocate on these issues. For example, in Case Study #1, a Partner who had conducted an observational, anthropological study of the nutritional value of meals in school canteens developed an analytical framework for integrating the motivations and actions of various actors [37]. The analysis revealed how important but diverse priorities—school funding, enterprise as welfare-promotion, and student health—underlying the different policies affecting school canteen operators undermined nutritional standards in student meals. The interactions of these policies were clear through the combined experiences of the different actors at the local level, but not through the viewpoints of any single actor or policy. The integrated approach in the case study enabled identification of key feedback loops that could be strengthened to increase the capacity and motivation of school canteen operators to provide healthy food options.

The PESB case studies had benefits beyond improved problem understanding. Several Partners requested further capacity building and engagement, having found the exercise valuable to their work. For example, one Partner initiated and funded a transdisciplinary workshop on campus sustainability, to extend insights from their case studies to other university actors. Additionally, the case studies provided compelling narratives which proved useful for Partners’ organisations, both internally to improve understanding, and externally to communicate effectively. This was evidenced among the Partners for Case Study #6, who initiated a subsequent systems analysis on another water supply and sustainability issue.

### Example: illustration of localisation, capacity-building, and values-as-indicators

A pair of case studies (#4 and #5) are examined in-depth here to illustrate the ways in which the case study process improved Partner understanding that led to new actions. These case studies focused on campus sustainability in the setting of a major Malaysian public university, unpacking how place-specific institutional structures and paradigms support or threaten educational and outreach efforts and sustainable land use choices [38, 39]. Partners originated from a botanic garden facility with a broad mission of conservation and education and from a closely associated grassroots initiative (alumni and student) for ecological engagement and volunteerism adopted by the university. The first case study examined drift in university support for the broad botanic garden mission, while the latter examined a project conducted by the grassroots initiative that contributed to the preservation of a rewilded land bank in the face of developmental pressures.

A wide body of literature establishes the positive contributions that green space and biodiversity make to health, especially mental health [40–42]. However, drivers that promote appreciation, preservation, and cultivation of green space are strongly place-based and heavily subject to local context, including socioeconomic conditions, developmental legacy and climate [43, 44]. Malaysia is a developing economy, in which income generation is a high priority. Land is a priceless resource in the city and the neoliberal developmental paradigm adopted worldwide over recent decades has resulted in the side-lining of green space conservation [45]. The intrinsic assumption in this paradigm is that undeveloped land constitutes an underutilised resource. Yet the modern-day reframing of development in terms of sustainability recognizes the value of green space. This is contained not only in SDG 15.9, calling for the integration of ecosystem and biodiversity values into national and local planning, but also in in SDG 11.7 which affirms the need to provide universal access to safe, inclusive and accessible, green and public spaces. University campuses and botanic gardens can contribute substantial institutional green space to a city [46], but most relevant examples come from well-resourced institutions in highly developed contexts.

These case studies explored the value to the university of maintaining or converting green spaces and the institutional values required to sustain urban green space efforts more broadly. The Partners’ initial framing of the problem was in terms of individual decision-makers and their values, and of institutional resource constraints. While the Partners have a degree of agency in addressing the issues at hand, the primary locus of decision-making authority lies elsewhere, contributing to a sense of disempowerment and uncertainty over the long-term viability of their efforts. Their engagement with the case studies was, in part, an attempt to advocate for their positions on these issues.

**Fig. 1:**
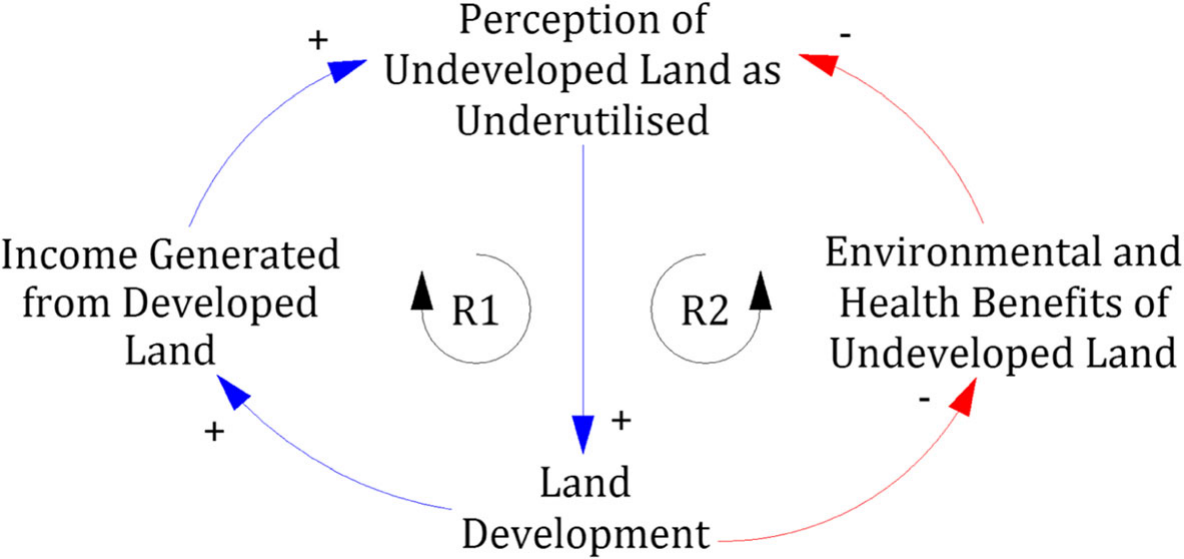
Partners discovered competing institutional narratives surrounding undeveloped university land, each driven by reinforcing feedback loops. The perception that undeveloped land is underutilised drives new development, which in turn generates income, reinforcing the perception that undeveloped land should be developed (R1). Conversely, when undeveloped land is perceived as valuable, low rates of development will preserve environmental and health benefits, and the experience of these benefits undermines the belief that undeveloped land is underutilised (R2). Figure is reproduced from Ong and Adikan (2018) [38].

A systems-level analysis shifted focus from personalities as guardians of values toward the influence of institutional structures and incentives in shaping institutional values. Partners attributed this to rigorous and repeated probing via the systems thinking process, which interrogated many underlying assumptions. For example, funding cuts to the botanic garden were originally ascribed to budgetary constraints stemming from reduction of public funding for the university. Further reflection revealed shifts in the university institutional priorities as the fundamental driver, as university budgetary constraints had merely accelerated funding cuts to the botanic garden, a trend that had begun long before. This revised conceptual model of events created a better appreciation of the various constraints decision-makers face and pointed toward institutional paradigms of undeveloped land as a core issue undermining support for biodiversity and greenspace initiatives (Fig. 1).

**Fig. 2:**
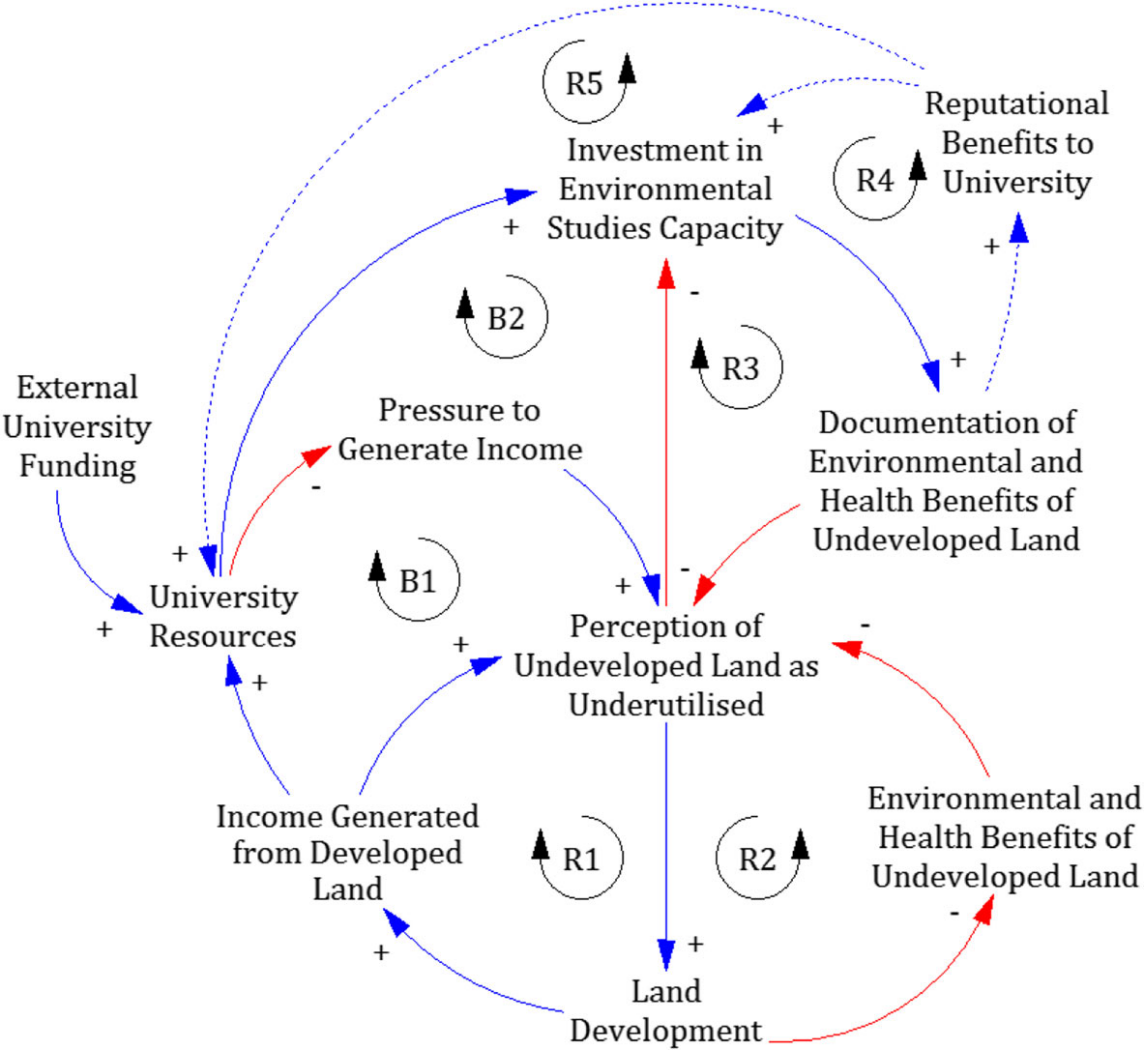
Partners identified several systemic relationships that strengthen or weaken the competing narratives. Availability of university resources, driven largely by external public funding, determines the level of pressure for income generation, which in turn can lead to land development to alleviate financial pressure (B1 loop). University efforts to document environmental and health benefits of undeveloped land have shaped perceptions of the land and encourage further investment in such studies (R3 loop); however, such efforts are also threatened by university funding limitations, which are further constrained by decisions to forego income generation to preserve undeveloped land (B2 loop). Partners identified reputational benefits to the university as useful leverage point if certain pathways (dotted arrows) could be developed and strengthened. Translation of documented environmental and health benefits of undeveloped university land into reputational benefits could reinforce university decisions to invest in environmental studies (R4). Reputational benefits could also be leveraged for income generation through edu-tourism and other means (R5). Figure is adapted from Ong and Adikan 2018 [38]

Whereas a general analysis of the issue of green space on campuses might focus on profit-vs-loss calculations, situating the issue within a unique place allowed for deeper consideration of the local socio-geographic context. The university’s rewilded land bank is a significant green space in a locality where nature is otherwise scarce. The engagement of student volunteers in this project provided low-cost capacity building through fieldwork training at a time when classroom-based practical sessions were threatened by severe funding cuts across the university. Choosing to maintain green space fostered good will with neighbourhood residents who would have been affected by the proposed development. These insights suggested that Partners need not only advocate ecological and sustainability causes, but also seek out the systemic feedbacks that shape institutional perspectives and values related to land use (Fig. 2).

The case study process provided Partners a space to reflect on the importance of underlying variables, causes, consequences, and feedbacks. In developing their conceptual models and narratives with the Systems Thinker, the Partners revisited everyday experiences. This reflection catalysed discovery of the interconnectedness of the issues and more importantly, their linkages to wider narratives of sustainability. Partners re-evaluated the value of various key performance indicators in directing focus to the actions necessary to advance the overarching mission. One such example was the identification of land use paradigms as a central issue. This led the grassroots initiative to invest effort in engaging the university in dialogue on how land is valued. A prior focus on green space and biodiversity indicators, which remain important outcomes, overlooked the importance of engaging institutional values driving university decision-making processes. The Partners also undertook a systems analysis (not described here) to evaluate how best to position themselves in ways that would allow them to do this over the long term. This illustrates the role that values-as-indicators in SDG localisation can play in drawing actors’ attention to critical processes that must be engaged to achieve desired outcomes [4, 5].

## Discussion

### Utility for SDG localisation

Systems thinking has often been used as a tool for scaling-up local interventions for improved health outcomes [47, 48]. Here, a different approach has been taken, using systems approaches for down-scaling and localisation, recognising that complexity and interconnections exist at all problem scales. Indeed, the PESB case study methodology was conceptualised to enhance decision-making, especially in the face of cross-sectoral issues that impact health and wellbeing, by improving capacity for systemic understanding and transdisciplinary communication. As a bottom-up process, it features minimal resource requirements. These design parameters make this approach uniquely suited for SDG localisation, in which complex and interconnected challenges particular to a specific place need to be addressed with locally available resources (Table 2).

**Table 2:** Example of SDG Localisation Problem that Can be Addressed with Case Study Methodology

While the Malaysia Sustainable Development Goals Voluntary National Review Report 2017 (Economic Planning Unit, Prime Minister’s Department, 2017) showed that Malaysia has made substantial overall progress toward SDG 3: Good Health and Well Being, sexual and reproductive health (SRH) nonetheless remains a concern. Contraceptives use and adolescents sexual and reproductive health remain difficult to operationalise due to diverse local gendered realities, culture and religion. Much of the shortcomings stem from problems in local implementation: SRH services are fragmented, with no clear mechanisms to coordinate and track progress. Common indicators and frameworks are necessary to coordinate action and leverage resources. Meaningful engagement with local communities has not been established particularly with the marginalised and underserved populations, including young people. Community members are perceived as passive recipients of FP and SRH programmes and services. There is a lack of understanding and buy-in from other crucial partners at the local level including the Youth and Sports Ministry, Ministry of Education, Department for Islamic Development, and local community leaders. Addressing SRH needs of unmarried, young people and adolescents remains controversial for most of the key partners. Here, we see the need for [1] systemic understanding to overcome fragmentation of efforts [2]; for sense-making tools to enable bottom-up approaches to generate contextually appropriate solutions; and [3] for powerful narratives that can challenge and shift deeply held paradigms. These needs in localisation are not unique to the challenges of FP and SRH, and the case study approach described herein attempts to address all three.	

In local SDG implementation, local indicators are important not only for measuring impacts, but for highlighting important processes that generate the desired outcomes. Indeed, systems thinking recognises that indicators are not just a measurement, but that the choice of indicators also changes system behaviour as indicators become targets and actors take actions accordingly [49]. This can be beneficial if indicators are well-aligned with actual goals but can be detrimental if there are pathways to achieve indicators that are not relevant—or even detrimental—to desired outcomes. The case study methodology enables actors to develop conceptual models of systems processes, enabling them to choose supportive indicators in a holistic manner that acknowledges critical relationships and system leverage points [25]. Key processes often include the inculcation and nurturing of values that support the SDG goals—enabling factors that are often neglected in indicator selection because of the difficulties in quantifying and standardising such subjective and place-specific variables [4, 5]. Systems approaches can enable and inform the process of re-examining accepted narratives, mitigating against path dependency so that indicators are not adopted merely because of prior usage [50].

The same improved conceptual models that enables better selection of local indicators also increases capacity to act. Systemic understanding is useful not only for identifying pathways and leverage points for achieving specific SDG targets, but also for identifying the potential unintended consequences of simultaneous SDG actions across different sectors and scales, as where efforts to achieve one SDG target reinforce or constrain efforts to achieve another [51]. Benefits are most apparent where multiple actors in sustainable development can be brought together in the development of a case study, with a simple systems model such as a CLD serving as an organizing principle for communication and relationship-building needed to achieve sustainable development.

Case studies are effective tools for advocating positions to policy-makers [52]. However, the complex messages and relationships frequently inherent in local operationalisation of the SDGs are often difficult to communicate in an accessible narrative. The CLDs used in the PESB case studies address this challenge, acting as metaphors that communicate complex ideas and relationships that are not easily communicated through words alone [21, 53]. The input of the System Thinker was important to effectively use CLDs in this manner. In general, Partners tended to push for greater detail and complexity in CLDs, to represent all the particularities of their case study. While additional complexity was useful in exploration and achieving a comprehensive understanding of the issue, the Systems Thinker generally advocated for simplification to make key relationships visually observable and comprehensible.

### Strengths and limitations

The PESB case study methodology is one of several ways (e.g., workshops, co-production of dynamic simulations, etc.) in which systems thinking and place-based research can be brought together in systems approaches and comes with particular strengths and weaknesses. The development of the case studies involved extended engagement, which allowed the Partners to use, practice, and develop the skills of creating and interpreting causal loop diagrams to a higher level than can be done in a short course or workshop. It is a flexible methodology with low costs, enabling its utilisation in a wide variety of challenges. As it relies heavily on Partners implicit knowledge, it does not require the extensive data that certain systems methodologies rely on—which is typically unavailable at local scales.

There are a number of limitations in the PESB case study methodology. It is an involved and potentially time-intensive process, and several prospective Partners declined to participate for this reason. Interpersonal connections are important in cross-disciplinary work [54], especially in small co-located projects [55], and were important in sustaining a multi-month collaborative process. The opportunity to showcase work or highlight issues at WUF9 was a key incentive for Partners. Finding or creating such opportunities may be important for obtaining Partner interest when there is not prior interpersonal connection or interest in systems methodologies. A second major limitation was the lack of representation from different stakeholder groups in most of the case studies, limiting the scope of perspectives that could have been otherwise achieved. It can be difficult to obtain the buy-in needed from different groups, a factor that implies significant interpersonal management challenges for the Systems Thinker. The problem of limited perspective was partially addressed by asking the Partners to reflect deeply on the motivations and paradigms of the other actors involved in their challenges.

The PESB case study methodology can complement other systems tools. It can serve as a catalyst for transdisciplinary systems thinking workshops by creating outputs that draw interest and can also be a way of sustaining learning and engagement with systems methodologies following an introductory workshop. The CLDs developed through the case studies are also a good starting point for low-order systems dynamics modelling that can further aid local decision-making [56]. The PESB case study methodology is not dependent upon other systems methodologies to achieve impact, however: improved understanding of causal linkages can in itself improve local decision-making for the SDGs, generating benefits for population health and wellbeing. The extent of impact in this methodology depends on the same conditions that other transdisciplinary engagement tools depend upon: long-term follow-up and commitment of resources to act upon insights generated via transdisciplinary understanding.

## Conclusion

A Systems Thinker engaged several local Partners to co-produce placially explicit, systems-based case studies, using systems approaches to develop conceptual models and narratives that describe and analyse local urban challenges that impact health. In addition to producing documents that visually communicated complex challenges, this provided a method, suitable for resource-poor contexts, for drawing out Partners’ implicit and tacit knowledge and placing it in a systems framework. This process improved Partners’ understanding of the challenges they faced, improving analysis and action.

Local decision-making is critical to operationalising the SDGs. This affects urban planning, delivery of health services, education, environmental management, and many other factors that shape population level health. While the complexities of interlinkages coupled with lack of resources makes localisation of the SDGs a daunting task, local actors have vast implicit and tacit knowledge that they can draw upon. The PESB case study methodology is a powerful way of enabling these actors to articulate this knowledge through conceptual models for synthesis, evaluation, and action. Such placially explicit models can be powerful tools to inform local decision-making and communication, increasing the likelihood of achieving desired outcomes in local actions toward the SDGs.

## Data Availability

Data sharing is not applicable to this article as no datasets were generated or analysed during the current study. The co-produced case studies can be accessed at http://www.thriveurban.info/wp-content/uploads/2018/02/SCHEMA-Case-Studies.pdf
